# Parental income in childhood and health outcomes across age groups: a register-based study from Norway

**DOI:** 10.1186/s12916-026-04735-w

**Published:** 2026-03-06

**Authors:** Bjørn-Atle Reme, Hans Fredrik Sunde, Fartein Ask Torvik, Jonas Minet Kinge, Bjørn Heine Strand, Jonathan Wörn

**Affiliations:** 1 Norwegian Institute of Public Health, Oslo, Norway; 2 Department of Health Management and Health Economics, University of Oslo, Blindern, P.O. box 1089, 0317 Oslo, Norway; 3 Promenta Research Center, Department of Psychology, University of Oslo, Oslo, Norway; 4 The Norwegian National Centre for Ageing and Health, Vestfold Hospital Trust, Tønsberg, Norway; 5 Department of Geriatric Medicine, Oslo University Hospital, Oslo, Norway

**Keywords:** Parental income, Primary care utilization, Health inequalities

## Abstract

**Background:**

Parental socioeconomic status is associated with health outcomes across age. However, the specific age-, gender-, and disease-related patterns linking parental income during early childhood to healthcare utilization across age remain poorly characterized. Enhanced understanding of these associations is essential to inform targeted interventions and improve health equity.

**Methods:**

This cross-sectional study analyzed primary care consultations for all Norwegian residents aged 10–59 in 2018 (*N* = 2,882,669), merged with parental income records from 1958 up until 2017. The analysis was restricted to GP consultations for diseases or disorders, excluding consultations for symptoms and complaints. Analyses were stratified by sex, age, and type of health problem. We also examined how adult income and education mitigate socioeconomic disparities in healthcare utilization.

**Results:**

Individuals from low parental income backgrounds had higher primary care utilization. In the lowest parental income quintile, females averaged 1.89 (SD 3.30) and males 1.24 (SD 2.65) consultations per year, compared to 1.60 (SD 2.94) and 1.00 (SD 2.21), respectively, in the highest quintile. Socioeconomic differences varied by age and disease type. Among females, the largest inequality occurred at age 24, with 1.97 (95% CI 1.89–2.05) consultations in the lowest quintile versus 1.17 (95% CI 1.11–1.23) in the highest. For males, inequality was largest at age 31, with 1.22 (95% CI 1.15–1.30) versus 0.78 (95% CI 0.74–0.83) consultations. Disease-specific differences showed the largest odds ratios for psychological (females: OR 1.54, 95% CI 1.51–1.57; males: OR 1.64, 95% CI 1.60–1.68) and endocrine/nutritional issues (females: OR 1.34, 95% CI 1.32–1.37; males: OR 1.35, 95% CI 1.31–1.38). Adjusting for adult education and income eliminated most disparities, except for musculoskeletal and endocrine/nutritional health problems.

**Conclusions:**

Lower parental income in childhood was associated with higher adult primary care use, particularly for psychological, musculoskeletal, and endocrine conditions, with heterogeneity by age and sex. These associations were attenuated when accounting for individuals’ own education and income, suggesting that policies that enhance educational attainment and economic opportunity can help reduce health inequalities.

**Supplementary Information:**

The online version contains supplementary material available at 10.1186/s12916-026-04735-w.

## Background

Despite universal health coverage and equal access to healthcare services in the Nordic countries, substantial socioeconomic gradients in health persist [1–4]. These gradients reflect complex mechanisms, including the bidirectional relationship between health and socioeconomic status. Income and education influence nutrition, housing, and lifestyle [5–9], while poor health can limit education, workforce participation, and engagement in health-promoting activities [10, 11]. Additionally, genetic factors come into play, whereby inherited genes have the potential to impact both the ability to achieve a high socioeconomic position and overall health [12, 13].

While health inequalities are well-studied, measuring them across life stages presents challenges. Health problems differ substantially at different life stages, making overall measures of inequality difficult to interpret and potentially obscuring important heterogeneities [14]. Understanding the extent of inequality in health and identifying the specific health problems driving these inequalities at each life phase is crucial for targeted interventions. This paper addresses these challenges by investigating health inequalities across age groups, from age 10 to 59, using primary healthcare utilization as a measure of health. Specifically, it aims to assess the age-, sex-, and disease-specific associations between parental income and healthcare utilization, and to explore the extent to which inequalities in health by parental income are mediated by individuals’ own socioeconomic status (education and personal income). In the Norwegian healthcare system, primary care providers act as the mandatory gatekeeper to specialist healthcare services. Accordingly, consultations are a comprehensive measure of population health that captures both actual morbidity and help-seeking behavior.

This study contributes to the literature by systematically mapping socioeconomic inequalities in healthcare utilization across age, from childhood to adulthood, in a universal healthcare setting. By combining population-wide register data with detailed measures of parental income, education, and personal income, we are able to disentangle the timing, nature, and potential pathways of these inequalities. Our approach highlights not only whether inequalities exist, but also which health problems and life stages are most affected. These insights are essential for informing targeted interventions and policies aimed at reducing health disparities in welfare states, where universal coverage alone has proven insufficient to eliminate socioeconomic gradients in health.

## Methods

### Data

This cross-sectional nationwide study used Norwegian registry data to examine health inequalities across childhood parental income, analyzing primary healthcare utilization among individuals aged 10–59 years by linking parental income to offspring healthcare utilization records.

The study used data from four linked administrative registers: the Norwegian Population Register, The National Income Register, the National Education Database, and the Norwegian Control and Distribution of Health Reimbursement Database (KUHR). The Norwegian National Population Register holds data on all individuals residing in Norway and contains a large set of demographic information, including a personal identification number, gender, date of birth, and the identification number of the parents. The National Income Register contains yearly records of taxable personal income for all Norwegian residents. The income measure includes all income from work and transfers that is subject to taxation. Data on educational attainment was drawn from the National Education Database, which contains annually updated records of completed education for Norwegian population. The Norwegian Control and Distribution of Health Reimbursement database (KUHR) includes nationwide data on all visits to primary healthcare. In this study, we focused on visits to general practitioners (GPs), which are the first line of healthcare provision and serve as gatekeepers for access to specialist healthcare. Hence, primary care better captures population-level demand for healthcare [15].

In Norway, there is universal health coverage except for a small copayment (appr. 20 EUROs) per GP consultation. The role of user-funded private healthcare and voluntary health insurance in healthcare financing, which is not reported to KUHR, is negligible in Norway. All visits to primary healthcare are registered with a diagnostic code—describing reasons for encounter—given according to The International Classification of Primary Care (ICPC-2). This study uses all registrations related to consultations by primary care physicians, with two exclusions. First, we excluded codes related to administration (A97), preventive care (A98), and unspecified health problem (A99). Second, we excluded consultations related to symptoms and complaints, as these are more likely to be subject to differences in inclination to seek help for less severe health problems.

The linkage across different registers was performed by Statistics Norway using personal identification numbers, ensuring high linkage quality. The personal identification numbers were replaced by a hashed ID before delivery to the National Institute of Public Health. The study was approved, and participant consent was waived by the Regional Committee for Medical and Health Research Ethics South-East Norway (REK, approval 2018/434). Participants were not required to provide informed consent for this study because it utilized regularly collected administrative register data.

### Study population

The study included all individuals between ages 10 and 59 registered as living in Norway in January 2018. The age range, especially the upper limit of 59 years, was chosen based on the income registry data available for this study, which covered incomes in Norway from 1958 up until 2019. This restriction ensured that our data included individuals with their parents’ income registered during their first 10 years of life (see Measures section for more details). Individuals with missing parental identity or income were excluded from the analysis. The study population was retrieved by combining the Norwegian Population Register and the National Income Register. See Additional file 1: Fig. S1 for a graphical presentation of the sample and exclusions.

### Measures

#### Measures of income and educational attainment

Parental income was retrieved from the national register containing pensionable earnings from the period when the index person was between 0 and 9 years old. To construct parental income quintiles, we first calculated the sum of both parents’ earnings each year, then calculated their income percentile each year, relative to other parents with children born in the same year. Then, the average of these percentiles over this 10-year period was calculated, and categorized into equally sized quintiles. When estimating participants’ own income quintile, we used registered income in 2018, stratified by birth year and gender.

Educational attainment was measured as the highest achieved level of education in 2018, according to the International Standard Classification of Education (ISCED). The ISCED was recoded as binary, where 1 corresponded to no higher-/university education (levels 0–5), and zero otherwise (levels 6–8).

### Measure of healthcare utilization

The study was based on primary care utilization. Specifically, we measured for each person in our analytical sample all in-person physical consultations with a GP in 2018. All Norwegian inhabitants are assigned a general practitioner (GP) but can also freely switch practitioner by using a secure web-based portal. For the GPs to be reimbursed for their care provision, their consultations and procedures must be registered in the Norwegian Control and Reimbursement Database (KUHR). The database also contains diagnostic information, which is coded according to the second edition of the International Classification of Primary Care (ICPC-2). ICPC-2 is divided into chapters, according to localization and type of health problem the patient has. This study reports from GP consultations related to more severe health problems, diseases or diagnoses—number 70 and up for each chapter. Note that we define as a consultation an inquiry for a specific diagnosis. Inquiries for different diagnoses during the same appointment are considered as multiple consultations.

Note that pregnancy is coded within the W-chapter (W76), although these consultations are not linked to diseases or disorders. The remaining codes in this chapter are more severe issues related to pregnancy.

### Statistical analyses

First, to describe overall patterns of healthcare utilization, we estimated the average number of GP consultations in 2018 within parental income quintiles 1, 2–4, and 5, separately by gender and age (10–59 years). Second, to explore differences across types of health problems, we estimated disease-specific odds ratios (OR) comparing the odds for those with low parental income (1st quintile) to the odds for those with high parental income (5th quintile), where each disease-type outcome was coded as a binary indicator (1 = ≥ 1 GP consultations; 0 = none). The odds ratios were estimated using logistic regressions, adjusting for age. Third, to explore variation across both age groups and disease types, we calculated the disease, age- and gender-specific difference in GP consultations between quintiles 1 and 5. Last, to assess the role of adult socioeconomic status in explaining disparities, we used logistic regressions to estimate disease-specific odds ratios, with and without adjustment for educational attainment and own income quintile. This analysis excluded individuals below age 30 to ensure that educational attainment and income measures were representing socioeconomic status in a meaningful way.

All analyses were run in R v.4.1.2.

## Results

The study sample consisted of *N* = 2,882,669 individuals. Out of these, 49% were female, and the average (sd) age was 34.2 (14.6). The average (sd) number of consultations in the quintile with the lowest parental income was 1.89 (3.30) among females, and 1.24 (2.65) among males. In the quintile with the highest parental income, the average was 1.60 (2.94) among females, and 1.00 (2.21) among males. There were 7,623,172 GP consultations, from which we excluded codes related to administrative contacts (4.0%). We then excluded consultations registered only with symptoms—not disease—codes (41.4%), resulting in an analytical sample of 4,160,042 GP consultations classified with disease codes.

**Fig. 1:**
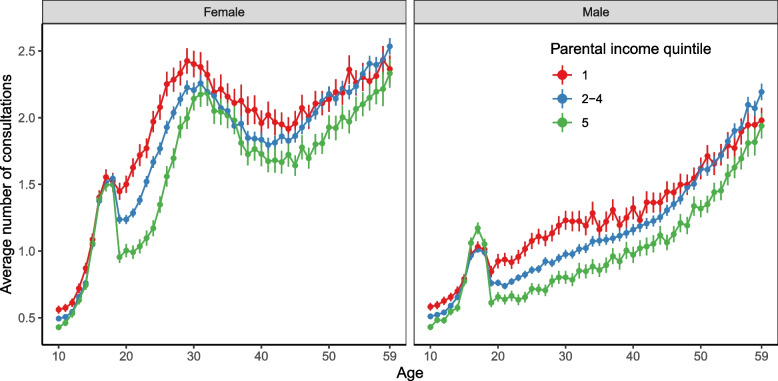
Average number of primary care consultations with general practitioner, by age, sex and parental income quintile during first 10 years of life. Quintile 1 (*N* = 576,553), quintiles 2–4 (*N* = 1,729,602), and quintile 5 (*N* = 576,514); error bars denote 95% confidence intervals. See Additional file 1: Tables S1A and S1B for the corresponding tabulations

The number of general practitioner consultations, across age and parental income quintiles, is shown in Fig. 1 (see Additional file 1: Tables S1A and S1B for corresponding tables). The average number of consultations increased between ages 10 and 30 for both sexes, but reached an earlier peak among women. For females, the peak difference between the lowest and highest quintile was at age 24, where those in the lowest parental income quintile had 1.97 consultations (95% CI 1.89–2.05), compared to 1.17 (95% CI 1.11–1.23) in the highest quintile, a statistically significant difference given the non-overlapping confidence intervals. For males, the peak difference occurred at age 31, with 1.22 consultations (95% CI 1.15–1.30) in the lowest quintile versus 0.78 (95% CI 0.74–0.83) in the highest quintile. The smallest absolute differences between the first and fifth parental income quintiles occurred at the extremes of our age range (ages 10–18 and 58–59), where confidence intervals showed minimal separation (see Additional file 1: Tables S1A, S1B, and Fig. S2). There were also differences in premature deaths across these income quintiles, 748 in the first quintile, compared to 466 in the fifth (see Additional file 1: Fig. S3 for more details).

With regard to disease-specific relative differences, when comparing the first and fifth parental income quintile (Table 1), the largest odds ratios for low parental income were found for psychological (OR = 1.54, 95% CI 1.51–1.57 for females; OR = 1.64, 95% CI 1.60–1.68 for males) and endocrine/nutritional health problems (OR = 1.34, 95% CI 1.32–1.37 for females; OR = 1.35, 95% CI 1.31–1.38 for males). For skin-related consultations, the odds ratio was significantly below 1 for both sexes, indicating a higher risk among those from the higher income quintile (see Additional file 1: Tables S2 and S3 for estimates stratified by age group, as well as combined prevalence estimates by disease types for income quintiles 1 and 5).

**Table 1:** Odds ratios for low parental income, 1st quintile compared to 5th, across different types of diagnoses, age adjusted

Sex	Diagnosis (ICPC2-chapter)	Odds ratios [95% CIs]
Female	A-General and unspecified	1.10 [1.07, 1.13]
	D-Digestive	1.25 [1.22, 1.28]
	K-Cardiovascular	1.20 [1.17, 1.23]
	L-Musculoskeletal	1.27 [1.24, 1.29]
	N-Neurological	1.15 [1.12, 1.18]
	P-Psychological	1.54 [1.51, 1.57]
	R-Respiratory	1.00 [0.98, 1.01]
	S-Skin	0.93 [0.92, 0.95]
	T-Endocrine and nutritional	1.34 [1.32, 1.37]
	W-Childbearing	0.96 [0.93, 0.98]
	Other	1.08 [1.07, 1.10]
Male	A-General and unspecified	1.08 [1.04, 1.11]
	D-Digestive	1.22 [1.19, 1.26]
	K-Cardiovascular	1.12 [1.09, 1.14]
	L-Musculoskeletal	1.33 [1.31, 1.36]
	N-Neurological	1.19 [1.15, 1.24]
	P-Psychological	1.64 [1.60, 1.68]
	R-Respiratory	0.99 [0.97, 1.00]
	S-Skin	0.91 [0.89, 0.93]
	T-Endocrine and nutritional	1.35 [1.31, 1.38]
	Other	1.00 [0.98, 1.02]

Notes: Odds ratios (OR) compare low parental income (1st quintile) with high parental income (5th quintile), estimated in the subsample restricted to individuals in parental income quintiles 1 and 5. Models included age and a binary indicator for low parental income; outcomes are coded as binary indicators (≥ 1 GP consultation for the category). “Other” combines ICPC-2 chapters B (Blood, Blood Forming Organs and Immune Mechanism), F (Eye), H (Ear), U (Urological), X (Female genital), Y (Male genital), and Z (Social problems)

**Fig. 2:**
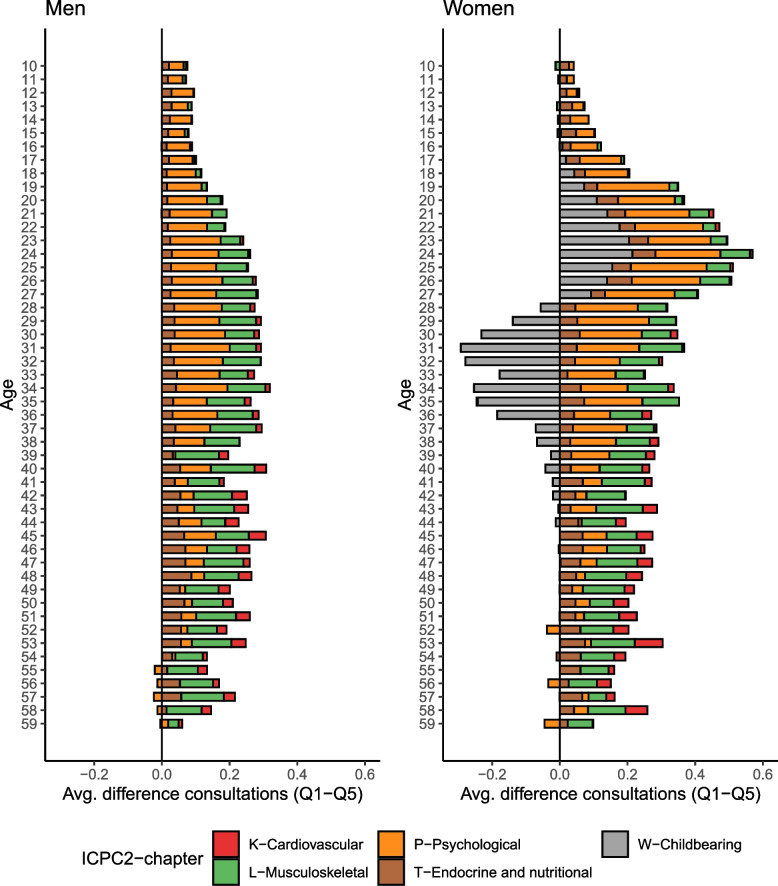
Difference between 1st and 5th quintile of parental income in primary care consultations, across sex, age and main categories of diagnoses. The included categories accounted for 52% of all visits. Respiratory conditions—the third most common category—were excluded owing to their infectious nature. See Additional file 1: Fig. S6 for results covering a broader set of health problems

When combining age-specific and disease-specific data for the most prevalent health problems where there were notable disparities (see Additional file 1: Figures S4 and S5), we found that large parts of the difference in the number of general practitioner consultations between the first and fifth income quintile arose from psychological and musculoskeletal health differences (Fig. 2). Note that we excluded respiratory (R) and skin (S) due to the lack of differences between the income quintiles (see Table 1, and Additional file 1: Fig. S6 for more disease types included). The difference in the average number of consultations for psychological problems was highest in the 20s and 30s; at age 25 for women (mean difference 0.22, 95% CI 0.18*–*0.27) and age 31 for men (mean difference 0.17, 95% CI 0.14*–*0.21). Differences in musculoskeletal health problems arose later and were at their highest at age 43 for women (mean difference 0.14, 95% CI 0.09*–*0.18) and age 37 for men (mean difference 0.14, 95% CI 0.10*–*0.17). There was also a higher number of healthcare consultations related to childbearing during the 20s among females from the lowest parental income quintile (mean difference 0.21, 95% CI 0.18*–*0.25). The pattern reversed in the 30s, where utilization was higher in the highest parental income quintile (mean difference − 0.29, 95% CI (− 0.36)*–*(− 0.23)). Additional file 1: Fig. S7 and S8 illustrate these inequalities for each disease type separately, and including base rates. Additional file 1: Table S4 includes results from the 20 most frequent individual diagnoses within the P (psychological), L (musculoskeletal), and T (endocrine/metabolism) chapters.

The socioeconomic differences in healthcare utilization based on parental income were substantially attenuated when adjusting for the individual’s own socioeconomic position, especially when adjusting for their income (Table 2). While the largest unadjusted differences were found for psychological health (OR = 1.31, 95% CI 1.27–1.34 for females; OR = 1.4, 95% CI 1.36–1.44 for males), these were practically non-existent when adjusting for income (OR = 0.97, 95% CI 0.95–1.00 for females; OR = 0.96, 95% CI 0.93–0.99 for males). However, differences related to musculoskeletal health problems and endocrine/metabolic health problems remained robust, also after adjusting for both education and income. See Additional file 1: Table S5 for a presentation of the distribution of parental income quintiles across individuals’ own income quintiles, and across participants’ educational attainment.

**Table 2:** Odds ratios for low parental income, 1st quintile compared to 5th, across different types of diagnoses and with and without adjustment for socioeconomic characteristics, age 30–59

Sex	Diagnoses (ICPC2 chapter)	Unadjusted Odds ratios [95% CI]	Adjusted: education Odds ratios [95% CI]	Adjusted: income Odds ratios [95% CI]	Adjusted: income and education Odds ratios [95% CI]
Female	A-General and unspecified	1.07 [1.03, 1.11]	0.99 [0.95, 1.03]	0.99 [0.95, 1.03]	0.95 [0.92, 0.99]
	D-Digestive	1.20 [1.16, 1.24]	1.10 [1.07, 1.14]	1.10 [1.06, 1.13]	1.06 [1.02, 1.09]
	K-Cardiovascular	1.20 [1.17, 1.23]	1.10 [1.07, 1.14]	1.13 [1.09, 1.16]	1.08 [1.05, 1.11]
	L-Musculoskeletal	1.26 [1.24, 1.29]	1.12 [1.10, 1.15]	1.16 [1.13, 1.18]	1.09 [1.06, 1.11]
	N-Neurological	1.09 [1.05, 1.12]	1.02 [0.98, 1.06]	0.97 [0.93, 1.00]	0.96 [0.92, 0.99]
	P-Psychological	1.31 [1.27, 1.34]	1.11 [1.08, 1.14]	0.97 [0.95, 1.00]	0.96 [0.93, 0.98]
	R-Respiratory	0.97 [0.95, 0.99]	0.93 [0.91, 0.95]	0.94 [0.92, 0.96]	0.92 [0.90, 0.93]
	S-Skin	0.91 [0.89, 0.93]	0.90 [0.88, 0.92]	0.89 [0.87, 0.91]	0.89 [0.87, 0.91]
	T-Endocrine and nutritional	1.21 [1.18, 1.24]	1.09 [1.06, 1.11]	1.08 [1.05, 1.11]	1.03 [1.01, 1.06]
	W-Childbearing	0.61 [0.59, 0.64]	0.75 [0.72, 0.78]	0.74 [0.71, 0.77]	0.81 [0.78, 0.84]
	Other**	1.04 [1.02, 1.06]	1.00 [0.98, 1.03]	1.00 [0.98, 1.02]	0.99 [0.97, 1.01]
Male	A-General and unspecified	1.10 [1.06, 1.15]	1.02 [0.98, 1.07]	1.01 [0.97, 1.06]	0.98 [0.93, 1.02]
	D-Digestive	1.18 [1.14, 1.22]	1.08 [1.04, 1.13]	1.08 [1.04, 1.12]	1.03 [0.99, 1.07]
	K-Cardiovascular	1.11 [1.08, 1.14]	1.02 [1.00, 1.05]	1.05 [1.03, 1.08]	1.00 [0.97, 1.03]
	L-Musculoskeletal	1.33 [1.30, 1.36]	1.13 [1.11, 1.16]	1.22 [1.19, 1.25]	1.09 [1.07, 1.12]
	N-Neurological	1.19 [1.13, 1.25]	1.06 [1.00, 1.11]	0.98 [0.93, 1.03]	0.95 [0.90, 1.00]
	P-Psychological	1.4 [1.36, 1.44]	1.17 [1.13, 1.20]	0.96 [0.93, 0.99]	0.93 [0.90, 0.96]
	R-Respiratory	0.97 [0.95, 0.99]	0.95 [0.93, 0.97]	0.93 [0.91, 0.95]	0.93 [0.90, 0.95]
	S-Skin	0.89 [0.87, 0.92]	0.92 [0.89, 0.94]	0.89 [0.86, 0.91]	0.91 [0.88, 0.93]
	T-Endocrine and nutritional	1.24 [1.21, 1.28]	1.11 [1.07, 1.14]	1.11 [1.08, 1.15]	1.05 [1.02, 1.08]
	Other**	1.00 [0.98, 1.03]	0.99 [0.96, 1.01]	0.96 [0.94, 0.99]	0.96 [0.94, 0.99]

Notes: Odds ratios (OR) compare low parental income (1st quintile) with high parental income (5th quintile), estimated in the subsample restricted to parental income quintiles 1 and 5, ages 30–59 years. Outcomes are coded as binary indicators of any GP consultation for the category. The unadjusted model includes age and the binary low–income indicator; the adjusted model additionally includes a binary indicator for any university education (ISCED level 6 or higher). **“Other” combines ICPC-2 chapters B (Blood, Blood-Forming Organs and Immune Mechanism), F (Eye), H (Ear), U (Urological), X (Female genital), Y (Male genital), and Z (Social problems)

## Discussion

In this nationwide study of nearly 2.9 million individuals and 4,160,042 general practitioner consultations, we demonstrate that parental income in early childhood is associated with patterns of primary care utilization decades later, even in a country with universal health coverage. We find that these disparities are not constant across age but peak in young adulthood for psychological problems and in midlife for musculoskeletal problems. While disparities persisted even after accounting for own education and income for musculoskeletal and endocrine/nutritional health problems, socioeconomic differences in psychological health problems largely disappeared when adjusting for the individual’s own adult education and income.

### Comparison to previous studies

Earlier studies, including from Norway and Sweden, have documented that lower childhood socioeconomic status is linked to poorer adult health and higher prevalence of chronic conditions, mental illness, and premature mortality [9, 16–21]. However, few studies have been able to track these associations across a wide age range and for multiple disease categories within the same population. Our results extend this evidence by showing that the strength and nature of socioeconomic disparities shift with age and diagnosis. The large differences in consultations for psychological problems among young adults from disadvantaged backgrounds resonate with previous reports of higher rates of depression, anxiety, and substance use in this group [22, 23]. By contrast, the emergence of musculoskeletal inequalities in middle age may reflect the cumulative impact of occupational strain, manual work, and health behaviors that are socially patterned.

Our findings also align with research on intergenerational health transmission emphasizing the importance of social mobility and cumulative disadvantage [24–26]. The fact that inequalities in psychological problems largely disappeared after adjustment for own education and income suggests that upward mobility can play a role in reducing early-life disadvantage. At the same time, alternative explanations cannot be ruled out given the cross-sectional nature of these associations. This moderating effect has been suggested in earlier Norwegian cohort studies, but our results show that it varies by diagnosis, highlighting the need for more differentiated policy responses.

### Mechanisms and interpretation

Several mechanisms likely explain our findings. First, the intergenerational transmission may be through socioeconomic position. Specifically, childhood socioeconomic disadvantage is associated with poorer educational outcomes, which in turn influence adult employment, income, and health. Our results for psychological problems fit this pattern—inequalities by parental income essentially disappeared after adjusting for respondents’ education and income, consistent with benefits of upward social mobility for mental health. Second, the transmission may partly be a result of cumulative exposure to adverse health behaviors such as smoking, unhealthy diet, and low physical activity—behaviors that are more common in disadvantaged groups—contributing to increased risks of metabolic and musculoskeletal disorders. Lastly, there may be genetic vulnerabilities transmitted through generations that cannot fully be mitigated by socioeconomic mobility.

We also observed higher primary care utilization related to childbearing during the 20s among females from the lowest parental income quintile. The pattern reversed in the 30s, where utilization was higher in the highest parental income quintile, likely reflecting the socioeconomic gradient in fertility timing. The small peak in consultations around ages 16–19 likely reflects institutional rules requiring medical certification to document sickness absence in upper secondary school.

Our findings should be interpreted within the context of Norway’s healthcare system with universal healthcare and modest copayments. Given the high accessibility, the higher utilization among low-income groups observed in this study likely reflects true health inequalities, whereas healthcare systems with higher user payments would show smaller differences due to financial barriers suppressing utilization among the poor. Still, the possibility of underutilization among low-income groups cannot be excluded.

### Strength and weaknesses

A major strength of this study is that it includes administrative records for the full population of individuals aged 10–59, residing in Norway in 2018 with known parental identity. Hence, there is limited selection and no recall bias.

Our study has several limitations. First, we use primary care utilization as a proxy for health. While this allows comprehensive population analysis, there is likely substantial variability across the population inclination to seek care. Differences in healthcare across socioeconomic backgrounds could hence to some extent be driven by differences in inclination, rather than differences in actual health. Another limitation involves confounding. Our study describes associations and cannot provide information on the mechanisms driving socioeconomic differences. There are several potential sources of confounding, or other unmeasured factors, that could bias our results. For example, although we adjust for age, this may not fully capture impacts stemming from early-life exposures (e.g., policy context, nutrition, diet, lifestyle, and environment). In addition, we cannot exclude the impact from unmeasured factors—such as birth cohort differences in health-seeking behavior—which may influence utilization independently of socioeconomic disparities. Together, these limitations mean that observed associations could partly reflect unmeasured differences across cohorts, care-seeking contexts and parental income groups rather than causal effects. Another limitation is that our analysis includes only individuals who survived until 2018. To the extent that survival prior to 2018 differs by parental income, this selective inclusion could introduce survivorship bias and distort the observed associations.

## Conclusions

Our study contributes to the understanding of socioeconomic disparities in health by showing that associations between childhood socioeconomic status and adult health are observable across a wide range of age groups, particularly pronounced for psychological, musculoskeletal, and endocrine health problems. But the associations were substantially attenuated, particularly for psychological health problems, when adjusting for own education and income. These findings suggest that policies promoting social mobility through education and income redistribution can reduce inequalities in some health domains. For policymakers and clinicians the message is clear: reducing intergenerational transmission of health disadvantages requires action well beyond equal access to healthcare, beginning in early life and sustained across several decades.

## Supplementary Information

Additional file 1: Figure S1 Flowchart of sample used in the analysis. Table S1A Average number of primary care consultations with general practitioner, by age, parental income quintile (Q) for females. Table S1B Average number of primary care consultations with general practitioner, by age, parental income quintile (Q) for males. Figure S2. Difference between 1st and 5th quintile of parental income in primary care consultations with general practitioners, across sex, age, all disease types. Figure S3. Cumulative number of deaths prior to 2018 for individuals that in 2018 would have been between 10 and 59 years old. Table S2 Odds ratios for low parental income, 1st quintile compared to 5th, across different types of diagnoses, by age groups. Table S3 Prevalence of diagnosis-types in quintile 1 and quintile 5. Figure S4. Share of total number of consultations for 1st and 5th quintile of parental income for different disease types. Figure S5A Average number of primary care consultations with general practitioner, by age, parental income quintile (1 and 5), across sex, age, and most prevalent disease types. Figure S5B Average number of primary care consultations with general practitioner, by age, parental income quintile (1 and 5), across sex, age, and most prevalent disease types. Figure S6. Difference between 1st and 5th quintile of parental income in primary care consultations, across sex, age and main categories of diagnoses. Figure S7. Difference (y-axis) and average (x-axis) between/for 1st and 5th quintile of parental income in primary care consultations, across sex, age groups, all disease types. Figure S8. Difference between 1st and 5th quintile of parental income in primary care consultations, across sex, age and main categories of diagnoses. Table S4 Odds ratios for low parental income, 1st quintile compared to 5th, across the most prevalent individual diagnoses, age adjusted. Table S5A Distribution of own and parental income quintile in the sample. Table S5B Distribution of own education and parental income quintile in the sample.

## Data Availability

The analysis relies on confidential individual-level data on health care utilization, education, and tax records from Norwegian administrative registers. Due to legal and privacy restrictions, these microdata are not publicly available. Researchers with approval from the Regional Committees for Medical and Health Research Ethics (REK) may apply for authorized access to the underlying data through the relevant custodian ( https://helsedata.no/en/).

## Abbreviations

CI: Confidence interval
GP: General practitioner
ICPC-2: International Classification of Primary Care, second edition
ISCED: International Standard Classification of Education
KUHR: Norwegian Control and Distribution of Health Reimbursement Database
OR: Odds ratio
REK: Regional Committee for Medical and Health Research Ethics
SD: Standard deviation
