# Supplementary material for: Parental income in childhood and health outcomes across age groups: a register-based study from Norway
Source: BMC Med. 2026 Mar 6;24:236. doi: 10.1186/s12916-026-04735-w (PMC13077859; doi:10.1186/s12916-026-04735-w)
Supplement: Supplementary file 1 — Additional file 1: Figure S1 Flowchart of sample used in the analysis. Table S1A Average number of primary care consultations with general practitioner, by age, parental income quintile (Q) for females. Table S1B Average number of primary care consultations with general practitioner, by age, parental income quintile (Q) for males. Figure S2. Difference between 1st and 5th quintile of parental income in primary care consultations with general practitioners, across sex, age, all disease types. Figure S3. Cumulative number of deaths prior to 2018 for individuals that in 2018 would have been between 10 and 59 years old. Table S2 Odds ratios for low parental income, 1st quintile compared to 5th, across different types of diagnoses, by age groups. Table S3 Prevalence of diagnosis-types in quintile 1 and quintile 5. Figure S4. Share of total number of consultations for 1st and 5th quintile of parental income for different disease types. Figure S5A Average number of primary care consultations with general practitioner, by age, parental income quintile (1 and 5), across sex, age, and most prevalent disease types. Figure S5B Average number of primary care consultations with general practitioner, by age, parental income quintile (1 and 5), across sex, age, and most prevalent disease types. Figure S6. Difference between 1st and 5th quintile of parental income in primary care consultations, across sex, age and main categories of diagnoses. Figure S7. Difference (y-axis) and average (x-axis) between/for 1st and 5th quintile of parental income in primary care consultations, across sex, age groups, all disease types. Figure S8. Difference between 1st and 5th quintile of parental income in primary care consultations, across sex, age and main categories of diagnoses. Table S4 Odds ratios for low parental income, 1st quintile compared to 5th, across the most prevalent individual diagnoses, age adjusted. Table S5A Distribution of own and parental income quintile in the sample. Table S5 [file 12916_2026_4735_MOESM1_ESM.pdf]

Supplementary Materials for

Parental Income in Childhood and Health Outcomes across Age Groups: A Register-Based Study from Norway

Table of contents **Page**

Figure S1 Flowchart of sample used in the analysis	2
Table S1A Average number of primary care consultations with general practitioner, by age, parental income quintile (Q) for females	3
Table S1B Average number of primary care consultations with general practitioner, by age, parental income quintile (Q) for males	4
Figure S2. Difference between 1st and 5th quintile of parental income in primary care consultations with general practitioners, across sex, age, all disease types	5
Figure S3. Cumulative number of deaths prior to 2018 for individuals that in 2018 would have been between 10 and 59 years old	5
Table S2 Odds ratios for low parental income, 1st quintile compared to 5th, across different types of diagnoses, by age groups	6
Table S3 Prevalence of diagnosis-types in quintile 1 and quintile 5	7
Figure S4. Share of total number of consultations for 1st and 5th quintile of parental income for different disease types	8
Figure S5A Average number of primary care consultations with general practitioner, by age, parental income quintile (1 and 5), across sex, age, and most prevalent disease types	9
Figure S5B Average number of primary care consultations with general practitioner, by age, parental income quintile (1 and 5), across sex, age, and most prevalent disease types	10
Figure S6. Difference between 1st and 5th quintile of parental income in primary care consultations, across sex, age and main categories of diagnoses	11
Figure S7. Difference (y-axis) and average (x-axis) between/for 1st and 5th quintile of parental income in primary care consultations, across sex, age groups, all disease types	12
Figure S8. Difference between 1st and 5th quintile of parental income in primary care consultations, across sex, age and main categories of diagnoses	13
Table S4 Odds ratios for low parental income, 1st quintile compared to 5th, across the most prevalent individual diagnoses, age adjusted	14
Table S5A Distribution of own and parental income quintile in the sample	15
Table S5B Distribution of own education and parental income quintile in the sample	15

Figure S1 Flowchart of sample used in the analysis

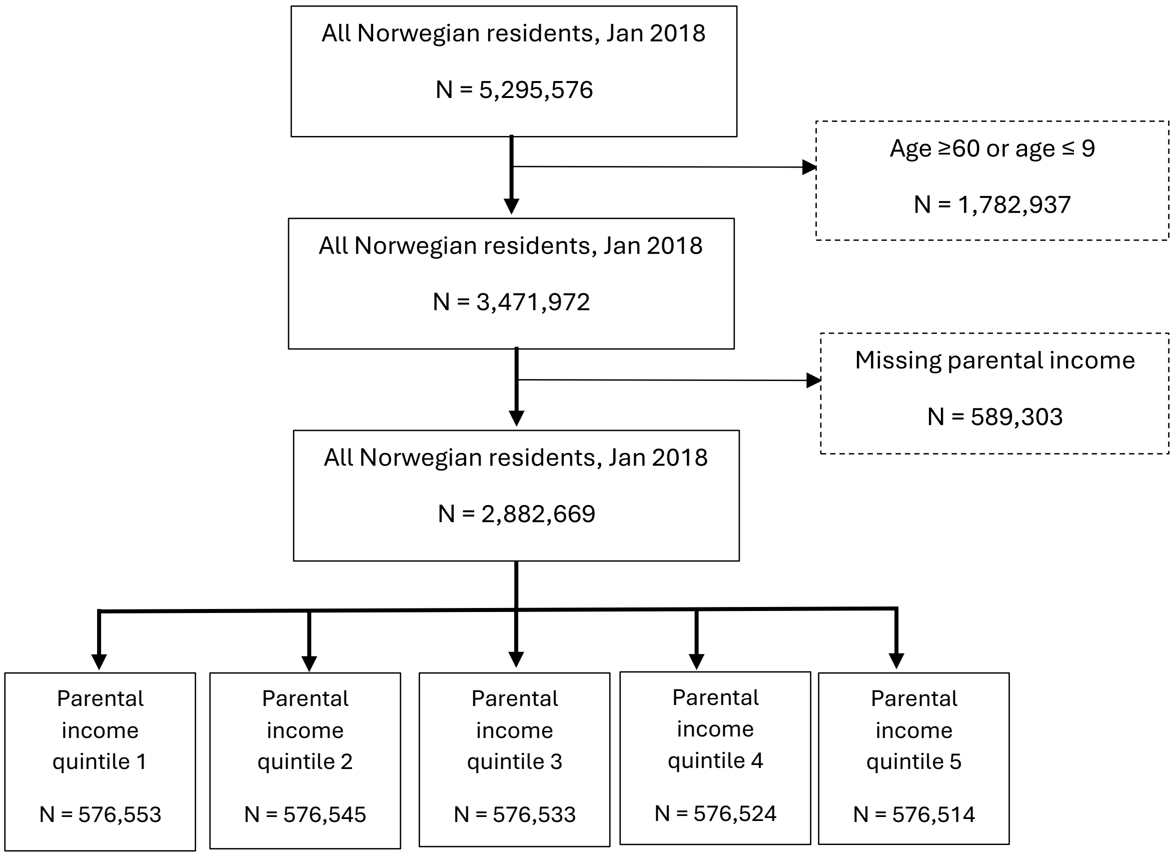

Table S1A Average number of primary care consultations with general practitioner, by age, parental income quintile (Q) for females

Age	Q1	Q2-Q4	Q5	Difference (Q1-Q5)
10	0.56(0.53-0.59)	0.49(0.48-0.51)	0.43(0.41-0.45)	0.13
11	0.57(0.54-0.6)	0.51(0.49-0.52)	0.46(0.44-0.49)	0.11
12	0.61(0.58-0.64)	0.54(0.53-0.56)	0.53(0.5-0.57)	0.08
13	0.72(0.68-0.76)	0.66(0.64-0.68)	0.63(0.6-0.66)	0.09
14	0.87(0.83-0.91)	0.76(0.74-0.78)	0.74(0.71-0.78)	0.13
15	1.09(1.04-1.13)	1.05(1.03-1.07)	1.06(1.01-1.1)	0.03
16	1.4(1.35-1.45)	1.38(1.35-1.41)	1.39(1.34-1.44)	0.01
17	1.55(1.5-1.61)	1.51(1.48-1.54)	1.5(1.45-1.55)	0.05
18	1.52(1.46-1.59)	1.54(1.51-1.58)	1.5(1.45-1.55)	0.02
19	1.45(1.38-1.51)	1.24(1.2-1.27)	0.95(0.91-1)	0.50
20	1.5(1.44-1.56)	1.24(1.2-1.27)	1(0.95-1.05)	0.50
21	1.63(1.56-1.69)	1.29(1.25-1.32)	0.99(0.94-1.04)	0.64
22	1.72(1.64-1.8)	1.38(1.34-1.42)	1.04(0.98-1.09)	0.68
23	1.77(1.7-1.84)	1.52(1.48-1.56)	1.1(1.04-1.15)	0.67
24	1.97(1.89-2.05)	1.67(1.62-1.71)	1.17(1.11-1.23)	0.80
25	2.08(1.99-2.17)	1.77(1.72-1.81)	1.35(1.29-1.41)	0.73
26	2.25(2.16-2.34)	1.93(1.88-1.98)	1.56(1.48-1.64)	0.69
27	2.29(2.2-2.38)	2.04(1.99-2.08)	1.7(1.62-1.77)	0.59
28	2.33(2.24-2.43)	2.14(2.09-2.19)	1.93(1.85-2.01)	0.40
29	2.43(2.33-2.52)	2.23(2.17-2.28)	2(1.91-2.08)	0.43
30	2.4(2.3-2.5)	2.21(2.15-2.26)	2.14(2.05-2.23)	0.26
31	2.38(2.27-2.49)	2.26(2.2-2.32)	2.18(2.08-2.27)	0.20
32	2.32(2.22-2.43)	2.19(2.14-2.25)	2.18(2.09-2.28)	0.14
33	2.19(2.08-2.29)	2.16(2.11-2.22)	2.05(1.95-2.15)	0.14
34	2.21(2.1-2.32)	2.07(2.01-2.13)	2.04(1.95-2.14)	0.17
35	2.16(2.06-2.26)	2.05(2-2.11)	2.02(1.92-2.11)	0.14
36	2.11(2.01-2.22)	1.94(1.88-1.99)	1.98(1.88-2.08)	0.13
37	2.13(2.01-2.25)	1.96(1.9-2.01)	1.81(1.72-1.9)	0.32
38	2.05(1.95-2.16)	1.85(1.8-1.9)	1.73(1.64-1.81)	0.32
39	2.06(1.95-2.17)	1.84(1.79-1.89)	1.77(1.68-1.85)	0.29
40	1.96(1.86-2.05)	1.84(1.78-1.89)	1.73(1.64-1.82)	0.23
41	2.02(1.92-2.12)	1.8(1.74-1.85)	1.67(1.59-1.76)	0.35
42	1.97(1.87-2.06)	1.81(1.76-1.86)	1.68(1.59-1.77)	0.29
43	1.95(1.86-2.04)	1.86(1.81-1.91)	1.67(1.58-1.75)	0.28
44	1.92(1.83-2)	1.83(1.78-1.88)	1.72(1.63-1.81)	0.20
45	1.96(1.87-2.05)	1.86(1.81-1.91)	1.64(1.56-1.72)	0.32
46	2.07(1.98-2.17)	1.93(1.88-1.98)	1.78(1.69-1.87)	0.29
47	2.01(1.92-2.1)	1.99(1.93-2.04)	1.7(1.62-1.77)	0.31
48	2.11(2.01-2.2)	2.04(1.99-2.09)	1.8(1.72-1.88)	0.31
49	2.11(2.02-2.2)	2.12(2.07-2.18)	1.81(1.73-1.89)	0.30
50	2.14(2.04-2.23)	2.18(2.12-2.23)	1.93(1.84-2.02)	0.21
51	2.19(2.1-2.29)	2.14(2.09-2.19)	1.92(1.83-2.01)	0.27
52	2.19(2.1-2.28)	2.22(2.16-2.27)	2(1.92-2.09)	0.19
53	2.36(2.25-2.47)	2.19(2.14-2.24)	1.97(1.88-2.06)	0.39
54	2.26(2.17-2.36)	2.24(2.18-2.29)	2.07(1.98-2.16)	0.19
55	2.3(2.2-2.41)	2.33(2.27-2.39)	2.1(2.01-2.19)	0.20
56	2.27(2.17-2.38)	2.41(2.35-2.46)	2.15(2.05-2.24)	0.12
57	2.31(2.21-2.41)	2.4(2.34-2.45)	2.19(2.1-2.29)	0.12
58	2.43(2.33-2.54)	2.43(2.37-2.48)	2.22(2.09-2.34)	0.21
59	2.36(2.26-2.47)	2.54(2.47-2.6)	2.33(2.23-2.44)	0.03

Table S1B Average number of primary care consultations with general practitioner, by age, parental income quintile (Q) for males

Age	Q1	Q2-Q4	Q5	Difference (Q1-Q5)
10	0.58(0.55-0.61)	0.51(0.5-0.52)	0.43(0.41-0.45)	0.15
11	0.59(0.56-0.62)	0.52(0.51-0.54)	0.48(0.46-0.51)	0.11
12	0.63(0.6-0.66)	0.54(0.52-0.56)	0.48(0.46-0.5)	0.15
13	0.66(0.62-0.69)	0.59(0.57-0.61)	0.55(0.52-0.57)	0.11
14	0.7(0.66-0.73)	0.65(0.63-0.67)	0.57(0.55-0.6)	0.13
15	0.79(0.75-0.83)	0.77(0.75-0.8)	0.77(0.74-0.81)	0.02
16	0.98(0.93-1.02)	0.97(0.94-0.99)	1.06(1.02-1.1)	-0.08
17	1.03(0.99-1.07)	1.01(0.99-1.03)	1.17(1.13-1.21)	-0.14
18	1.01(0.96-1.06)	0.99(0.97-1.02)	1.05(1.01-1.09)	-0.04
19	0.85(0.8-0.89)	0.76(0.74-0.78)	0.61(0.58-0.65)	0.24
20	0.92(0.87-0.98)	0.76(0.74-0.79)	0.66(0.62-0.69)	0.26
21	0.94(0.89-0.99)	0.74(0.71-0.76)	0.64(0.6-0.67)	0.30
22	0.92(0.87-0.97)	0.77(0.75-0.8)	0.66(0.63-0.7)	0.26
23	0.96(0.9-1.01)	0.8(0.77-0.83)	0.63(0.6-0.67)	0.33
24	1.02(0.96-1.07)	0.82(0.8-0.85)	0.65(0.61-0.69)	0.37
25	1.08(1.02-1.14)	0.86(0.83-0.89)	0.72(0.67-0.76)	0.36
26	1.11(1.05-1.17)	0.87(0.84-0.89)	0.71(0.67-0.75)	0.40
27	1.1(1.04-1.15)	0.92(0.89-0.95)	0.71(0.66-0.75)	0.39
28	1.13(1.07-1.2)	0.91(0.88-0.94)	0.78(0.73-0.82)	0.35
29	1.19(1.12-1.26)	0.95(0.91-0.98)	0.8(0.75-0.85)	0.39
30	1.23(1.16-1.3)	0.98(0.94-1.01)	0.8(0.75-0.85)	0.43
31	1.22(1.15-1.3)	0.98(0.94-1.01)	0.78(0.74-0.83)	0.44
32	1.22(1.15-1.3)	1.01(0.98-1.05)	0.85(0.79-0.91)	0.37
33	1.19(1.12-1.26)	1.02(0.99-1.06)	0.85(0.8-0.9)	0.34
34	1.28(1.2-1.37)	1.07(1.03-1.11)	0.88(0.83-0.94)	0.40
35	1.16(1.1-1.23)	1.08(1.04-1.12)	0.86(0.81-0.91)	0.30
36	1.22(1.14-1.3)	1.09(1.05-1.12)	0.89(0.84-0.95)	0.33
37	1.31(1.23-1.39)	1.1(1.06-1.14)	0.96(0.9-1.02)	0.35
38	1.2(1.13-1.26)	1.11(1.07-1.15)	0.92(0.86-0.98)	0.28
39	1.25(1.17-1.33)	1.14(1.09-1.18)	1(0.94-1.07)	0.25
40	1.32(1.24-1.41)	1.16(1.12-1.2)	0.97(0.91-1.03)	0.35
41	1.23(1.16-1.3)	1.19(1.14-1.23)	1.02(0.96-1.08)	0.21
42	1.37(1.28-1.45)	1.21(1.17-1.25)	1.03(0.97-1.1)	0.34
43	1.36(1.29-1.44)	1.23(1.19-1.27)	1.05(0.99-1.12)	0.31
44	1.37(1.29-1.44)	1.25(1.21-1.29)	1.12(1.05-1.19)	0.25
45	1.44(1.37-1.52)	1.31(1.26-1.35)	1.06(1.01-1.12)	0.38
46	1.44(1.37-1.51)	1.35(1.31-1.39)	1.13(1.07-1.19)	0.31
47	1.5(1.42-1.58)	1.39(1.35-1.43)	1.21(1.14-1.28)	0.29
48	1.5(1.42-1.57)	1.48(1.44-1.52)	1.19(1.13-1.26)	0.31
49	1.55(1.47-1.63)	1.5(1.46-1.55)	1.34(1.26-1.41)	0.21
50	1.62(1.54-1.7)	1.62(1.57-1.66)	1.32(1.25-1.38)	0.30
51	1.71(1.63-1.79)	1.61(1.57-1.66)	1.35(1.29-1.41)	0.36
52	1.66(1.57-1.74)	1.67(1.62-1.71)	1.44(1.37-1.51)	0.22
53	1.72(1.64-1.8)	1.72(1.67-1.76)	1.45(1.38-1.53)	0.27
54	1.79(1.69-1.89)	1.82(1.77-1.87)	1.57(1.49-1.65)	0.22
55	1.77(1.68-1.86)	1.9(1.85-1.96)	1.63(1.55-1.71)	0.14
56	1.9(1.8-1.99)	1.92(1.87-1.97)	1.69(1.61-1.78)	0.21
57	1.95(1.85-2.04)	2.1(2.04-2.15)	1.81(1.71-1.91)	0.14
58	1.95(1.85-2.04)	2.07(2.02-2.13)	1.82(1.72-1.92)	0.13
59	1.98(1.89-2.07)	2.19(2.13-2.26)	1.94(1.84-2.03)	0.04

Figure S2. Difference between 1st and 5th quintile of parental income in primary care consultations with general practitioners, across sex, age, all disease types

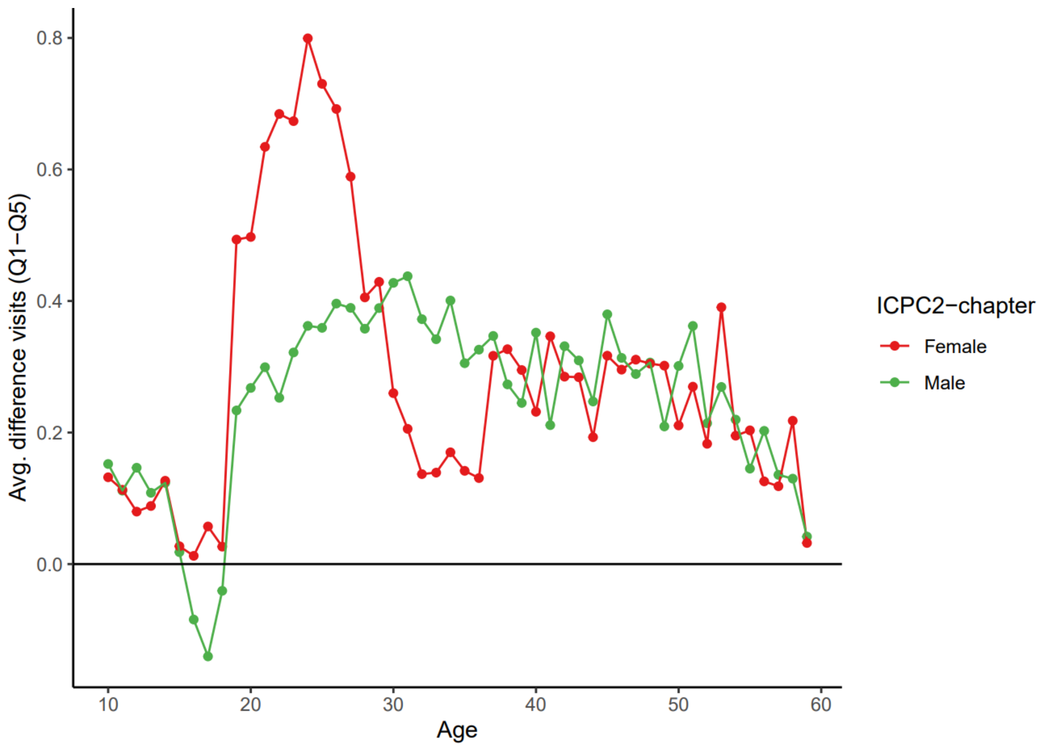

Figure S3. Cumulative number of deaths prior to 2018 for individuals that in 2018 would have been between 10 and 59 years old

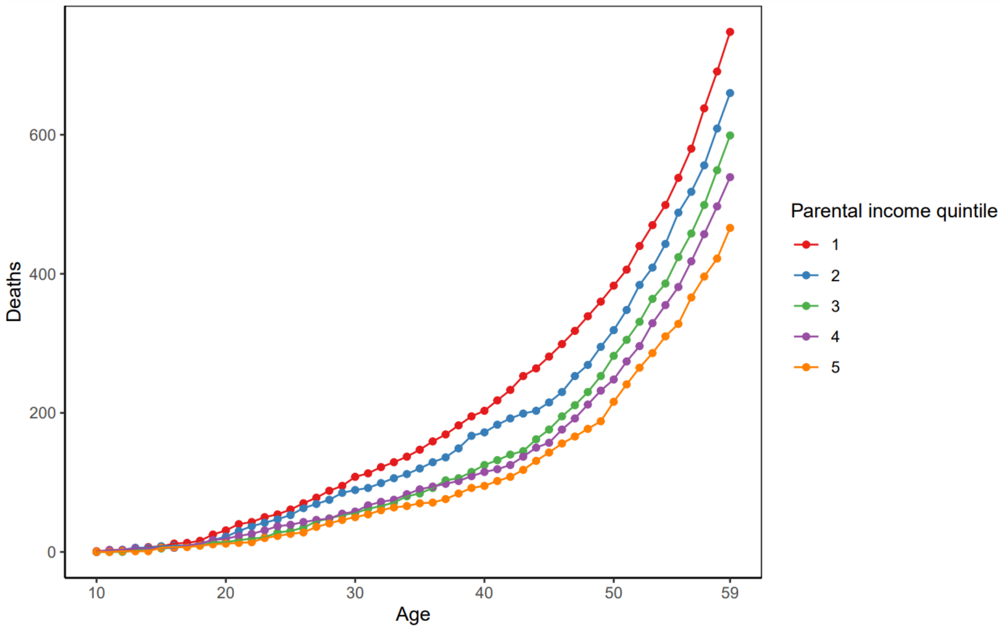

Notes. The figure uses the population register to calculate the cumulative sum of premature deaths for the different quintiles of parental income. These are individuals that would have been in the sample, had they not deceased. In sum the number of deaths were 748 in the first quintile; 539 in the second; 599 in the fourth; 539 in the fifth; 466 in the sixth.

Table S2 Odds ratios for low parental income, 1st quintile compared to 5th, across different types of diagnoses, by age groups

Sex	Diagnosis (ICPC2-chapter)	Age group 10-19	Age group 20-29	Age group 30-39	Age group 40-49	Age group 50-59
Female	A-General and unspecified	1.03 [0.97,1.09]	1.25 [1.18,1.33]	1.13 [1.06,1.21]	1.08 [1.02,1.15]	1.02 [0.96,1.08]
	D-Digestive	1.26 [1.18,1.34]	1.37 [1.3,1.45]	1.2 [1.13,1.27]	1.19 [1.12,1.26]	1.22 [1.16,1.29]
	K-Cardiovascular	1.04 [0.88,1.23]	1.26 [1.15,1.38]	1.19 [1.11,1.28]	1.24 [1.18,1.31]	1.18 [1.13,1.22]
	L-Musculoskeletal	0.99 [0.94,1.04]	1.6 [1.53,1.68]	1.52 [1.45,1.58]	1.31 [1.26,1.35]	1.12 [1.09,1.16]
	N-Neurological	1.15 [1.08,1.23]	1.36 [1.28,1.45]	1.29 [1.22,1.37]	1.11 [1.05,1.17]	0.9 [0.85,0.96]
	P-Psychological	2.02 [1.91,2.13]	2.02 [1.95,2.11]	1.65 [1.58,1.72]	1.26 [1.2,1.31]	1.05 [1,1.1]
	R-Respiratory	0.89 [0.87,0.92]	1.22 [1.18,1.26]	0.99 [0.96,1.03]	0.93 [0.9,0.96]	0.99 [0.95,1.02]
	S-Skin	0.95 [0.91,0.98]	0.98 [0.94,1.01]	0.92 [0.88,0.96]	0.93 [0.89,0.97]	0.89 [0.85,0.92]
	T-Endocrine and nutritional	2.06 [1.91,2.21]	1.72 [1.63,1.82]	1.35 [1.28,1.41]	1.24 [1.19,1.3]	1.12 [1.08,1.16]
	W-Childbearing	4.77 [3.74,6.08]	1.46 [1.4,1.52]	0.61 [0.58,0.63]	0.81 [0.72,0.91]	0.75 [0.42,1.33]
	Other	1.08 [1.04,1.12]	1.2 [1.17,1.24]	1.09 [1.05,1.13]	1.06 [1.03,1.1]	0.98 [0.95,1.02]
Male	A-General and unspecified	0.98 [0.92,1.05]	1.12 [1.05,1.2]	1.15 [1.06,1.24]	1.15 [1.06,1.23]	1.03 [0.96,1.1]
	D-Digestive	1.13 [1.05,1.2]	1.46 [1.38,1.56]	1.27 [1.19,1.35]	1.16 [1.09,1.23]	1.12 [1.06,1.19]
	K-Cardiovascular	1.23 [1.04,1.45]	1.23 [1.11,1.35]	1.17 [1.09,1.26]	1.24 [1.18,1.29]	1.03 [1.00,1.07]
	L-Musculoskeletal	1.06 [1.01,1.11]	1.62 [1.54,1.69]	1.55 [1.48,1.62]	1.33 [1.28,1.37]	1.22 [1.18,1.26]
	N-Neurological	1.06 [0.98,1.15]	1.38 [1.27,1.51]	1.3 [1.19,1.43]	1.25 [1.15,1.35]	1.06 [0.98,1.15]
	P-Psychological	2.1 [1.98,2.22]	2.04 [1.95,2.13]	1.75 [1.67,1.84]	1.4 [1.33,1.47]	1.05 [1,1.11]
	R-Respiratory	0.87 [0.85,0.9]	1.22 [1.18,1.27]	1.02 [0.98,1.06]	0.92 [0.89,0.96]	0.97 [0.93,1]
	S-Skin	0.94 [0.9,0.98]	0.93 [0.89,0.97]	0.93 [0.89,0.98]	0.88 [0.84,0.92]	0.87 [0.84,0.91]
	T-Endocrine and nutritional	2.18 [1.99,2.39]	1.74 [1.61,1.88]	1.45 [1.36,1.55]	1.39 [1.32,1.45]	1.1 [1.06,1.14]
	Other	0.97 [0.93,1.02]	1 [0.96,1.05]	1.03 [0.98,1.08]	1 [0.95,1.05]	0.99 [0.95,1.03]

Notes: Odds ratios (OR) compare low parental income (1st quintile) with high parental income (5th quintile), estimated in the subsample restricted to individuals in parental income quintiles 1 and 5. Models were run separately for age groups 10-19, 20-29, 30-39, 40-49 and 50-59. A binary indicator was used for low parental income; outcomes are coded as binary indicators (≥1 GP consultation for the category). “Other” combines ICPC-2 chapters B (Blood, Blood Forming Organs and Immune Mechanism), F (Eye), H (Ear), U (Urological), X (Female genital), Y (Male genital), and Z (Social problems).

Table S3 Prevalence of diagnosis-types in quintile 1 and quintile 5. This shows the prevalence of different types of diagnoses for a subsample of quintile 1 and 5, corresponding to analysis presented in Table 1 in the main analysis.

**Sex**	**Diagnoses (ICPC2 chapter)**	**Prevalence**
Female	A-General and unspecified	3.8 %
	D-Digestive	4.4 %
	K-Cardiovascular	4.1 %
	L-Musculoskeletal	11.2 %
	N-Neurological	4.0 %
	P-Psychological	8.3 %
	R-Respiratory	16.8 %
	S-Skin	10.3 %
	T-Endocrine and nutritional	6.8 %
	W-Childbearing	4.3 %
	Other	1.8 %
Male	A-General and unspecified	2.8 %
	D-Digestive	3.5 %
	K-Cardiovascular	4.9 %
	L-Musculoskeletal	9.4 %
	N-Neurological	2.0 %
	P-Psychological	5.8 %
	R-Respiratory	12.3 %
	S-Skin	7.4 %
	T-Endocrine and nutritional	4.6 %
	Other	0.9 %

Figure S4. Share of total number of consultations for 1st and 5th quintile of parental income for different disease types

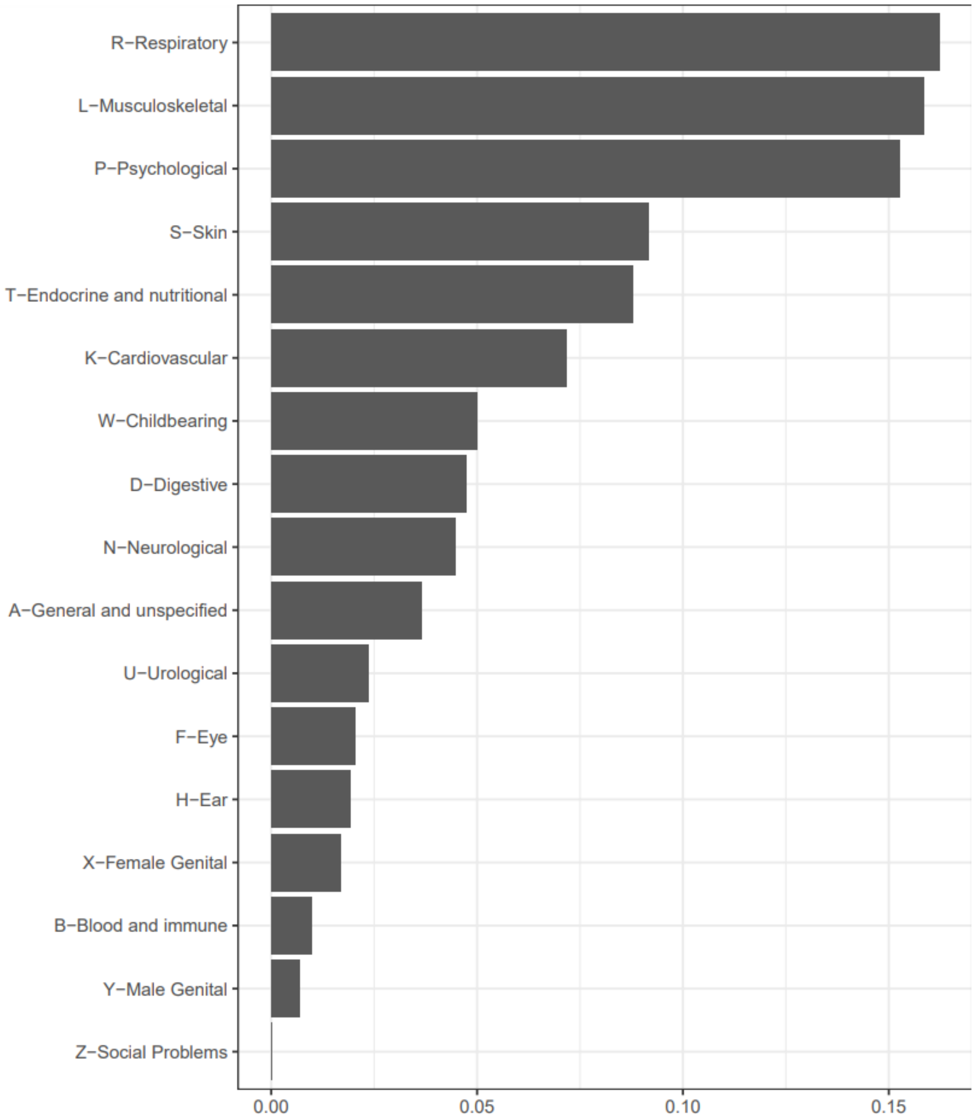

Figure S5A Average number of primary care consultations with general practitioner, by parental income quintile (1 and 5), across sex and age, for the most prevalent disease types

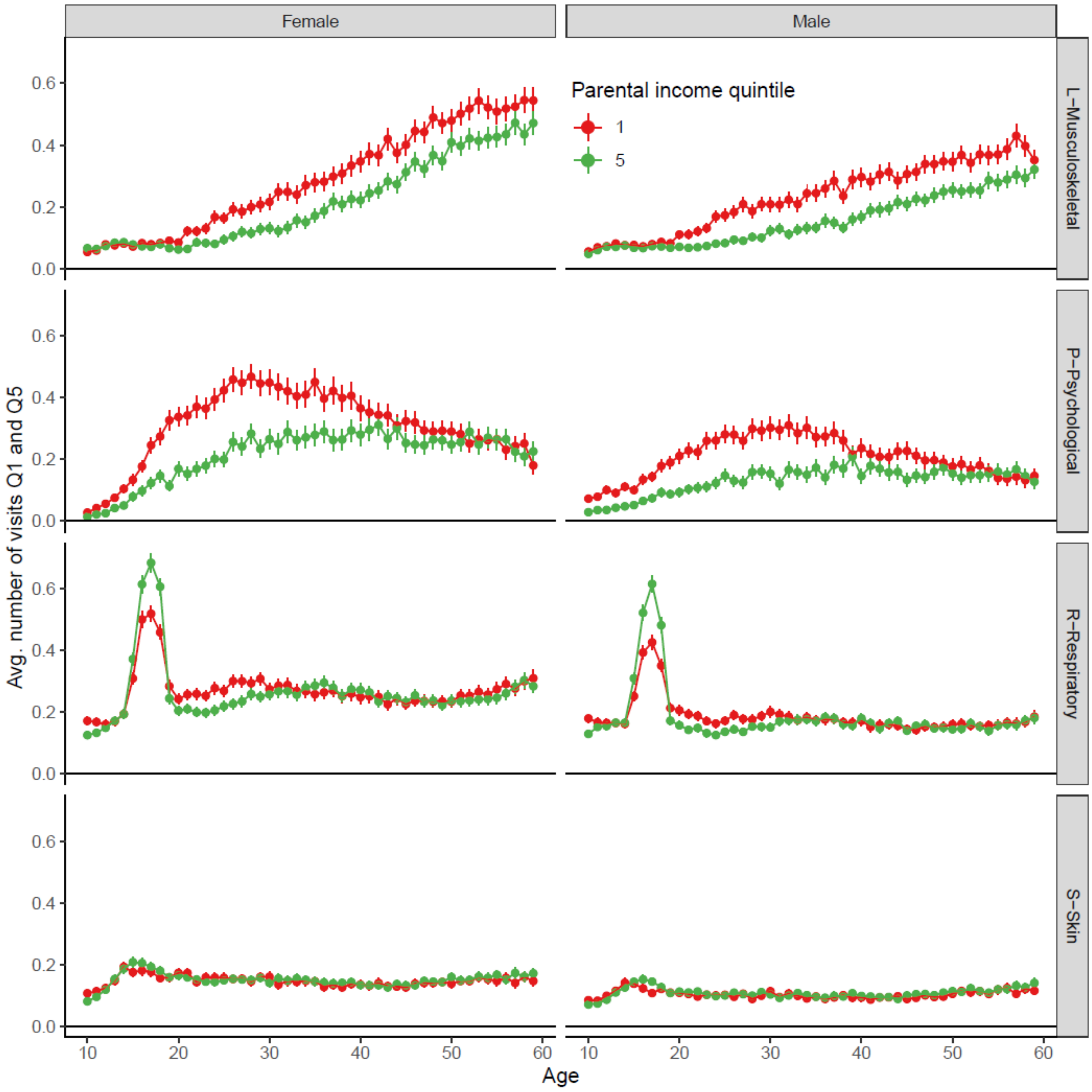

Notes: Average number of consultations and to the general practitioner in 2018 for each age and parental income quintiles 1 and 5. 95% CIs are indicated by error bars.

Figure S5B Average number of primary care consultations with general practitioner, by parental income quintile (1 and 5), across sex and age, for the most prevalent disease types

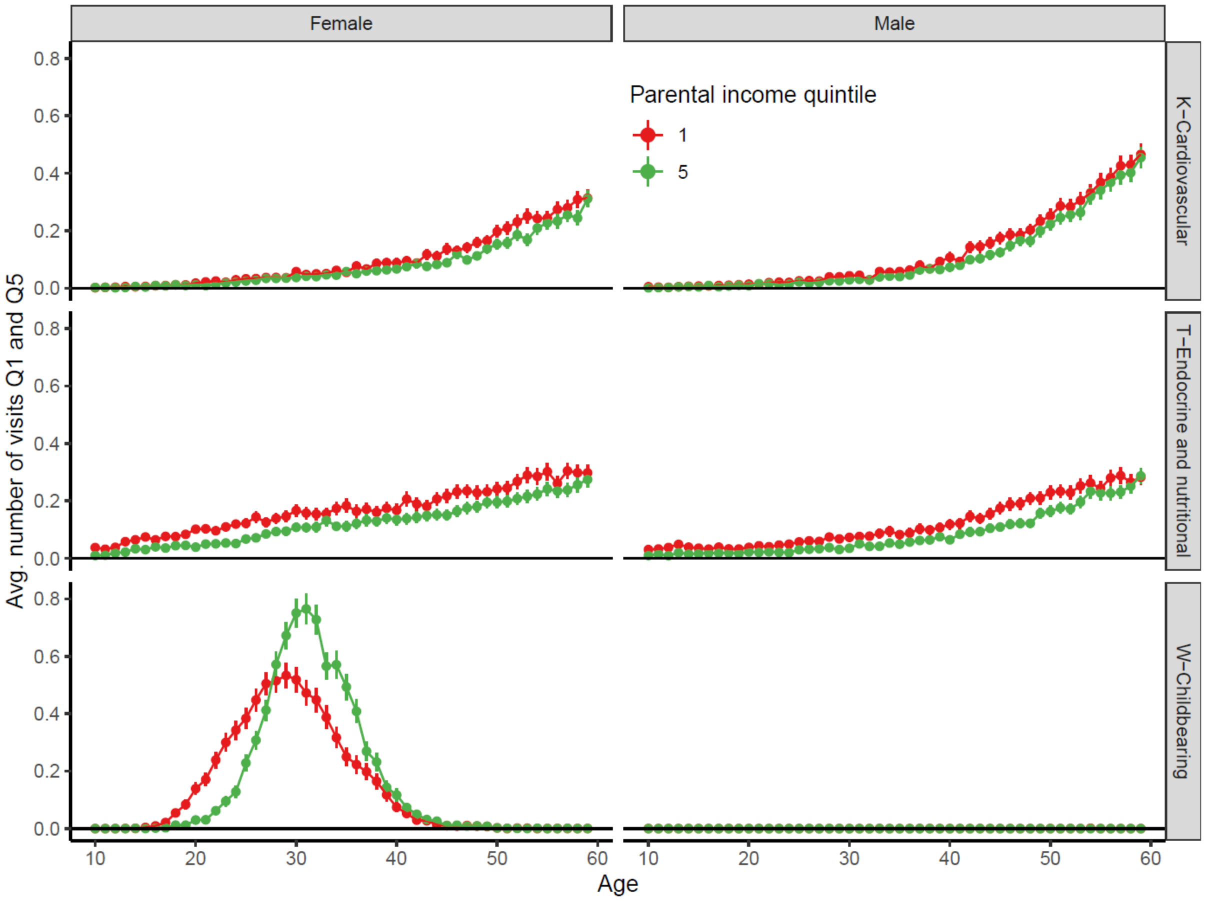

Notes: Average number of consultations and to the general practitioner in 2018 for each age and parental income quintiles 1 and 5. 95% CIs are indicated by error bars.

Figure S6. Difference between 1st and 5th quintile of parental income in primary care consultations, across sex, age and main categories of diagnoses

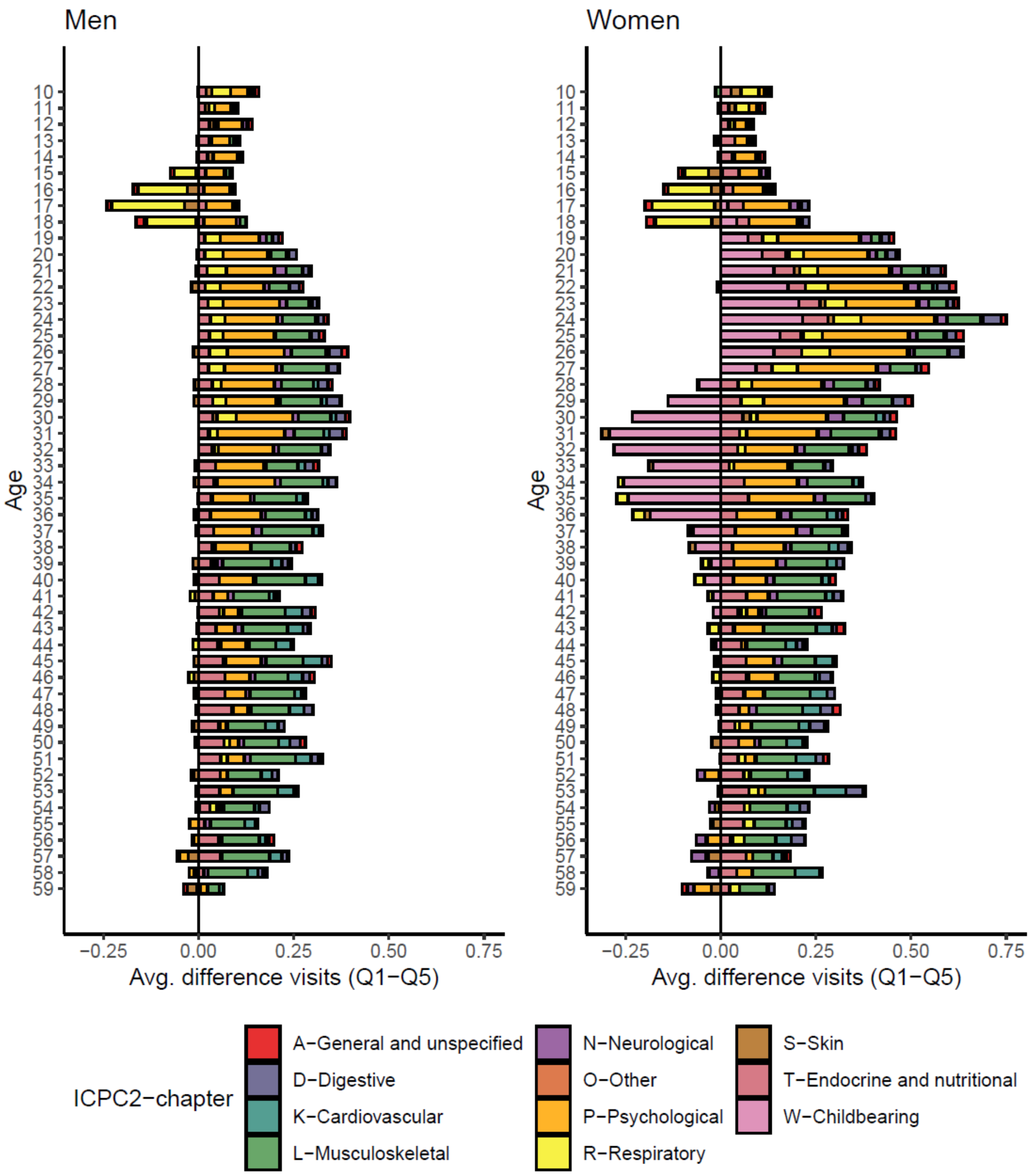

Notes: The difference between the first and fifth parental income quintile in number of consultations, by age, sex and the most relevant types of health problems.

Figure S7. Difference (y-axis) and average (x-axis) between/for 1st and 5th quintile of parental income in primary care consultations, across sex, age groups, and disease types

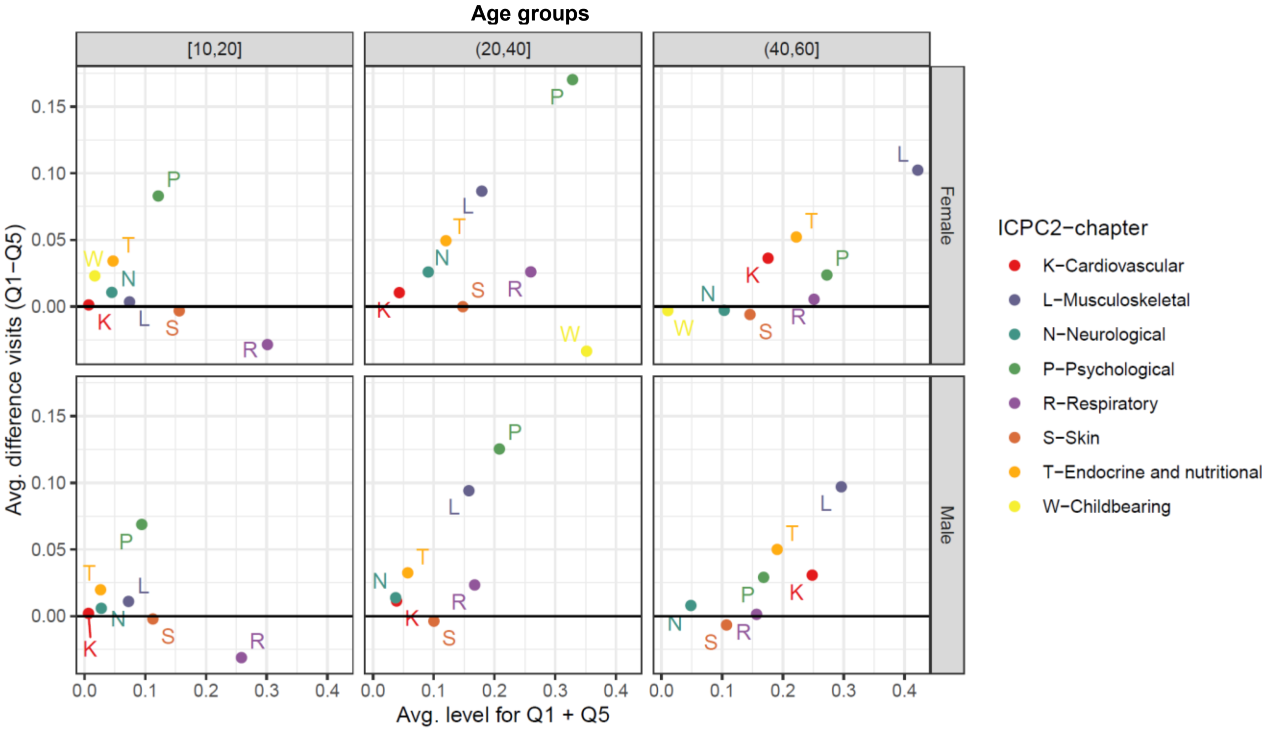

Notes: The difference between the first and fifth parental income quintile in number of consultations (y-axis) and average level of consultations for the first and fifth parental income quintile (x-axis). by age groups. sex and the most relevant types of health problems. To make the figure readable. The included disease types were limited to the chapters of ICPC2 that had a maximum average across all groups above 0.1 consultations.

Figure S8. Difference between 1st and 5th quintile of parental income in primary care consultations across sex, age, and main categories of diagnoses

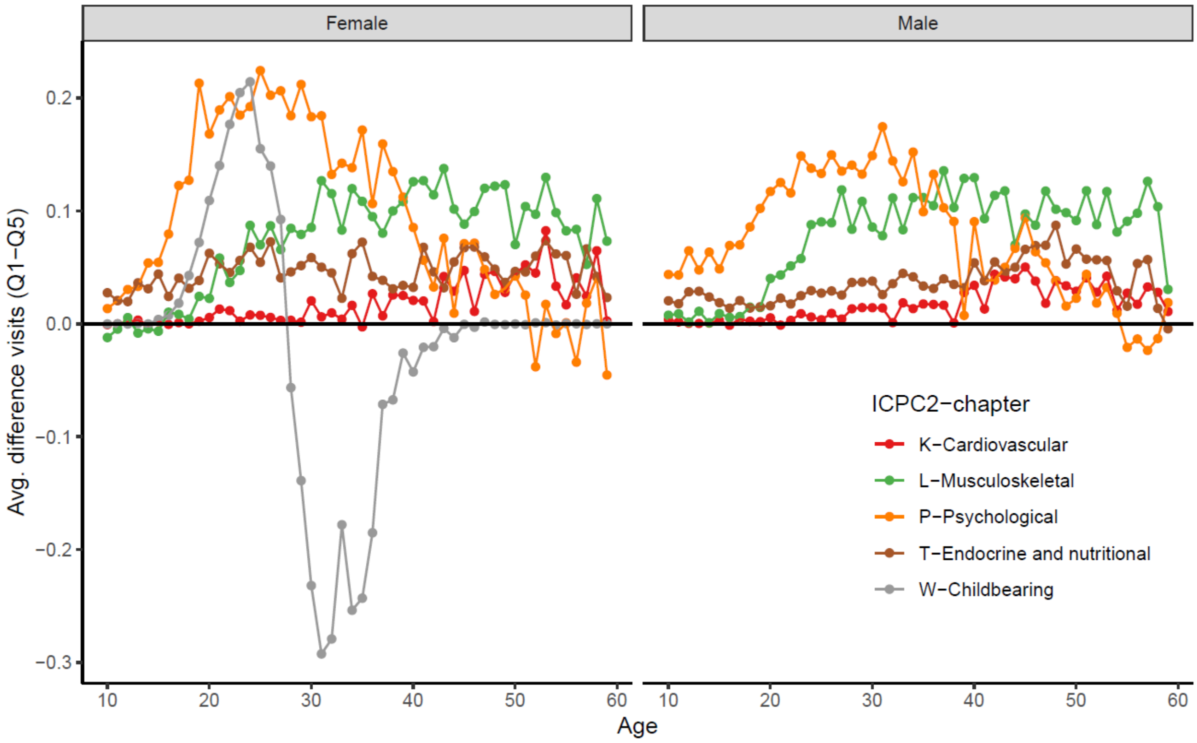

Notes: The difference between the first and fifth parental income quintile in number of consultations, by age, sex and the most relevant types of health problems.

Table S4 Odds ratios for low parental income: 1st quintile compared to 5th, across the most prevalent individual diagnoses. age adjusted. The table reports the odds ratio and 95% CI between quintile 1 and 5 for the 20 most frequent individual diagnoses from ICPC-2 chapters P (psychological), L (musculoskeletal), and T (endocrine/metabolism) observed in GP consultations during the study period. The diagnoses are sorted alphabetically.

Sex	Diagnoses	OR [95%CI]
Female	L76-Fracture: Not specified	1.158 [1.062,1.264]
	L81-Injury musculoskeletal NOS	1.158 [1.092,1.227]
	L83-Neck syndrome	1.384 [1.302,1.471]
	L84-Back syndrome without radiat pain	1.513 [1.432,1.599]
	L86-Back syndrome with radiating pain	1.603 [1.524,1.685]
	L87-Bursitis/tendinitis/synovitis NOS	1.194 [1.152,1.238]
	L88-Rheumatoid/seropositive arthritis	1.303 [1.214,1.399]
	L92-Shoulder syndrome	1.493 [1.427,1.562]
	L93-Tennis elbow	1.281 [1.192,1.376]
	L99-Musculoskeletal disease other	1.228 [1.163,1.296]
	P74-Anxiety disorder/anxiety state	1.532 [1.465,1.601]
	P76-Depressive disorder	1.381 [1.345,1.419]
	P79-Phobia/compulsive disorder	1.659 [1.536,1.791]
	P81-Hyperkinetic disorder	2.099 [1.973,2.233]
	T83-Overweight	2.000 [1.885,2.123]
	T86-Hypothyroidism/myxoedema	1.041 [1.001,1.083]
	T90-Diabetes non-insulin dependent	1.848 [1.742,1.961]
	T91-Vitamin/nutritional deficiency	1.459 [1.399,1.521]
	T93-Lipid disorder	0.974 [0.914,1.037]
	T99-Endocr/metab/nutrit disease other	1.331 [1.24,1.43]
Male	L76-Fracture: Not specified	1.321 [1.223,1.428]
	L81-Injury musculoskeletal NOS	1.247 [1.184,1.314]
	L83-Neck syndrome	1.751 [1.624,1.89]
	L84-Back syndrome without radiat pain	1.579 [1.496,1.666]
	L86-Back syndrome with radiating pain	1.593 [1.514,1.676]
	L87-Bursitis/tendinitis/synovitis NOS	1.131 [1.084,1.179]
	L88-Rheumatoid/seropositive arthritis	1.347 [1.227,1.479]
	L92-Shoulder syndrome	1.534 [1.459,1.614]
	L93-Tennis elbow	1.352 [1.261,1.45]
	L99-Musculoskeletal disease other	1.228 [1.152,1.308]
	P74-Anxiety disorder/anxiety state	1.658 [1.565,1.757]
	P76-Depressive disorder	1.401 [1.354,1.449]
	P79-Phobia/compulsive disorder	1.623 [1.493,1.763]
	P81-Hyperkinetic disorder	2.016 [1.914,2.123]
	T83-Overweight	1.763 [1.639,1.897]
	T86-Hypothyroidism/myxoedema	1.263 [1.15,1.388]
	T90-Diabetes non-insulin dependent	1.588 [1.516,1.663]
	T91-Vitamin/nutritional deficiency	1.583 [1.488,1.684]
	T93-Lipid disorder	0.905 [0.859,0.954]
	T99-Endocr/metab/nutrit disease other	1.442 [1.323,1.572]

Table S5A Distribution of own and parental income quintile in the sample

	Parental income quintile 1	Parental income quintile 2	Parental income quintile 3	Parental income quintile 4	Parental income quintile 5
**Income quintile 1**	25.5 %	20.2 %	18.5 %	17.7 %	18.1 %
**Income quintile 2**	22.8 %	21.4 %	20.1 %	18.8 %	16.9 %
**Income quintile 3**	20.2 %	20.9 %	20.7 %	20.1 %	18.1 %
**Income quintile 4**	17.6 %	19.9 %	20.8 %	21.3 %	20.5 %
**Income quintile 5**	13.9 %	17.6 %	19.9 %	22.1 %	26.4 %

Notes: Each row sums to one. The table shows within each income quintile of the index person (rows). The share of parents that belonged to each parental income quintile (columns).

Table S5B Distribution of own education and parental income quintile in the sample

Education level of index person	Parental income quintile 1	Parental income quintile 2	Parental income quintile 3	Parental income quintile 4	Parental income quintile 5
Basic<br>(ISCED 1–2)	27.0 %	20.8 %	17.4 %	13.7 %	8.3 %
Upper secondary<br>(ISCED 3–4)	40.5 %	41.4 %	38.9 %	34.2 %	24.4 %
Short tertiary:<br>Vocational/Bachelor (ISCED 5–6)	26.0 %	30.3 %	33.8 %	37.9 %	41.4 %
Long tertiary:<br>Master/PhD (ISCED 7–8)	5.9 %	7.2 %	9.6 %	14.1 %	25.7 %

Notes: Each column sums approximately to one (less than 1% missing own education were not included). The table shows within each income quintile of the index person (rows). The share of parents that belonged to each parental income quintile (columns).
